# Virus-derived gene transfer agents benefit host cells by providing templates for DNA repair

**DOI:** 10.1371/journal.pbio.3001874

**Published:** 2022-11-08

**Authors:** Andrew S. Lang

**Affiliations:** Department of Biology, Memorial University, St. John’s, Newfoundland and Labrador, Canada

## Abstract

The potential benefits to bacteria of producing gene transfer agents have long been speculated. This Primer explores a new study in PLOS Biology showing that DNA transfer by these phage-like elements allows host cells with damaged DNA to perform DNA repair and survive.

## Teaser

The potential benefits of producing gene transfer agents (GTAs) have long been speculated. A new study in *PLOS Biology* shows that DNA transfer by these phage-like elements allows cells with DNA damage to perform DNA repair and survive.

Gene transfer agents (GTAs) are a fascinating group of genetic elements that have been discovered in both the bacterial and archaeal domains. They look like tailed bacteriophages, but, unlike phages, they are incapable of self-transmission. This is because they are not capable of packaging and transferring all of the genes required to produce the phage-like GTA particles. For example, the model GTA produced by the bacterium *Rhodobacter capsulatus*, called RcGTA, packages approximately 4 kb of linear double-stranded DNA (dsDNA) [1], whereas the gene cluster that encodes most of the RcGTA proteins spans approximately 14 kb [2]. GTAs package DNA from throughout the producing cells’ genomes and this DNA can then be transferred to other cells. It is possible to imagine how this gene transfer might be beneficial to a recipient cell, but the fact that the cells that make GTAs die has led to difficulties in explaining how the organism benefits overall given this cost. There are other known instances where death of individual cells can benefit the population, such as during abortive infection where a phage-infected cell dies to prevent phage replication and spread [3]. But the potential benefit from GTA production is not as obvious. They must be doing something for the organisms that make them, otherwise they would not be maintained—but what? Various possible benefits have been proposed, but proof has been absent thus far. In this issue, Gozzi and colleagues [4] have experimentally demonstrated that a GTA is produced by the bacterium *Caulobacter crescentus* and, most importantly, shown that it benefits the bacterium by mediating DNA repair within the population.

The idea that GTAs could be beneficial by their act of mediating gene transfer goes back to shortly after their discovery [5] and was proposed by the original GTA discoverer and pioneer Barry Marrs, who wrote “we favor considering this system as a bacterial mechanism that exists because of the selective advantage which the capability for genetic exchange might confer” [6]. Subsequent discoveries of other GTAs revealed they have evolved independently multiple times in disparate groups of microbes, ranging from methanogens to pathogenic spirochetes to free-living purple non-sulfur bacteria. Of particular note is the conservation of GTA genes within some lineages of the bacterial class alphaproteobacteria [7]. The extant members of these GTA gene-containing organisms would have diverged from a common ancestor hundreds of millions of years ago. That would seem to be enough time to rid themselves of the genes if they were not beneficial, but the genes remain. The group of bacteria where GTA genes are the most widely and highly conserved are abundant in natural environments, particularly in the oceans where they have been estimated to make up as much as 40% of the total microbial community in some locations. Therefore, it is possible that some of the viral particles observed in natural environments are actually GTAs.

Other findings that have supported the notion that GTAs must provide some benefit to the producing organism include some of the details about how their production is regulated. In *R*. *capsulatus*, there are multiple cellular regulatory pathways that affect RcGTA production. One of these is population density-based quorum sensing, which increases RcGTA gene expression and release of the particles into the environment [8]. Therefore, more GTAs are made when there are more cells around. Moreover, quorum sensing and a regulatory protein essential for GTA gene expression also affect the ability of cells to interact with GTAs and uptake the packaged DNA. These cellular regulatory systems affect the synthesis of extracellular polysaccharides on the surface of the cell to which RcGTA binds and competence proteins that transfer the dsDNA that entered the periplasm from GTAs to the cytoplasm as single-stranded DNA (ssDNA) [9]. This renders the transferred DNA insensitive to cytoplasmic restriction enzymes. The overall result is that there is a coordinated division of behaviors within the population of cells where <3% become activated to make GTAs and lyse while the remainder of the population develops the ability to acquire DNA from the particles [9].

**Fig 1:**
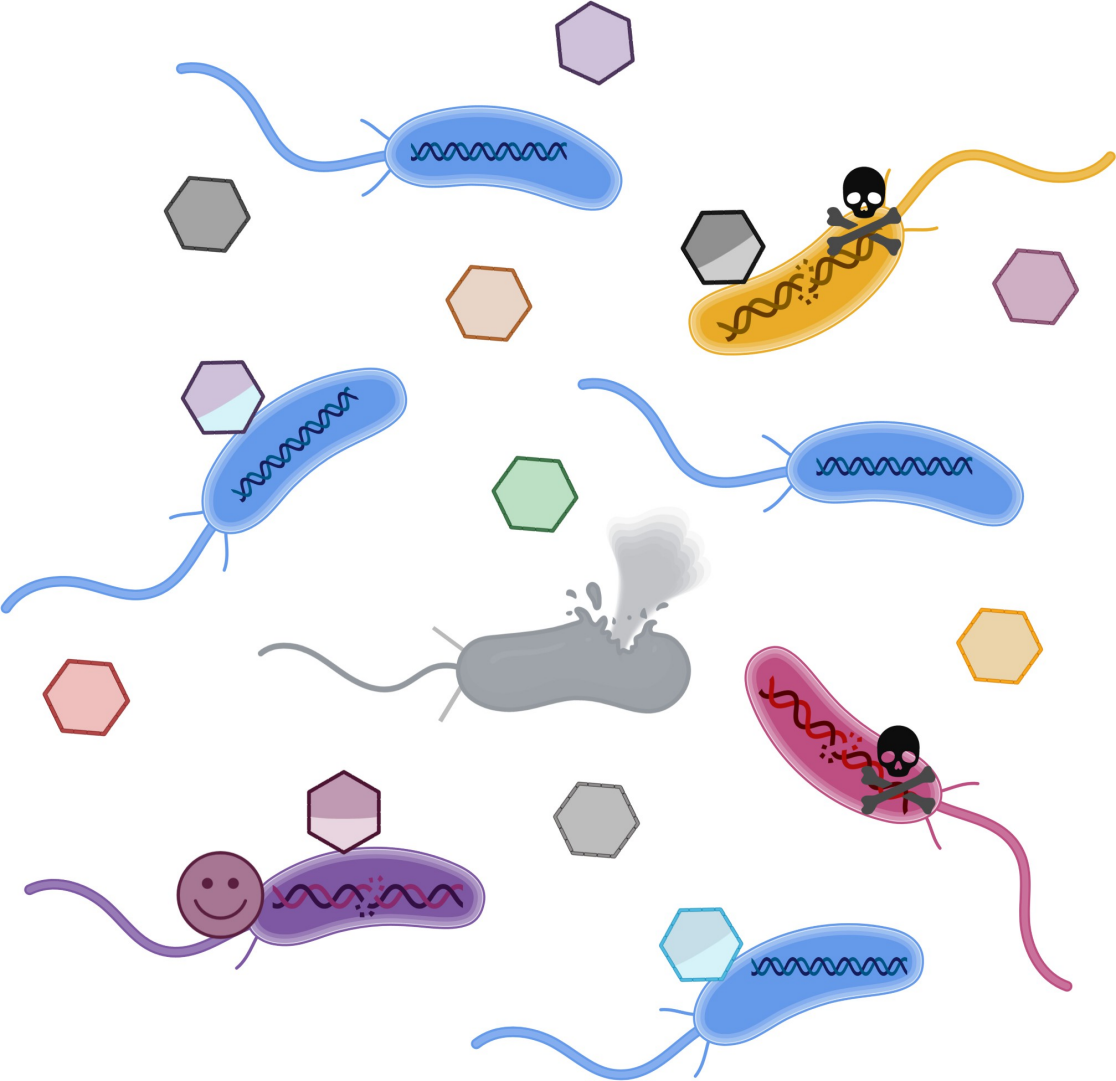
GTAs transfer DNA to recipient cells that can be used for DNA repair. GTA production by a cell in the population (lysed gray cell) results in release of packaged genomic fragments into the environment where each GTA contains a different fragment from the cell’s genome. These GTAs can then transfer the packaged DNA to recipient cells. The work by Gozzi and colleagues [4] shows that this transferred DNA helps recipient cells by acting as a substrate for recombination-mediated DNA repair. The pink, purple, and yellow cells have suffered DNA damage and will die without receiving the appropriate fragment of DNA from a GTA to act as a substrate for repair. Lucky cells (e.g., the purple cell) receive a DNA fragment that corresponds to the region requiring repair and survive. Cells that receive DNA from a GTA, but not the appropriate region to facilitate the needed DNA repair (e.g., the yellow cell), and cells that do not receive any DNA from a GTA (e.g., the pink cell) will die. The blue cells have not suffered DNA damage and can survive with or without receiving DNA from GTAs. Created with BioRender.com.

In the current work, Gozzi and colleagues [4] demonstrated that the *C*. *crescentus* GTA packages approximately 8 kb of DNA from throughout the genome. The associated GTA gene cluster is approximately 14 kb, and thus this GTA meets the non-self-transmissible criterion. GTA production is not apparent in wild-type cells in laboratory conditions, but they identified a regulatory protein in whose absence GTA production is elevated and detectable. They showed that strains that produce more GTAs have increased survival through prolonged starvation. The authors then considered and tested 3 potential explanations by which GTA production might accomplish this benefit. They ruled out the acquisition of beneficial alleles and release of nutrients into the environment by the cells that lyse to release GTAs as underlying mechanisms. Instead, it was the transfer of DNA into the recipient cells that provided the survival benefit, but it was the use of this transferred DNA as a template for recombination-based DNA repair that was the key to the increased survival. GTAs were shown to allow cells to recover from DNA damage induced by UV light and chemical agents and specific introductions of double-strand breaks (Fig 1). The GTAs were also shown to function as a “public good” as coincubation of GTA-producing cells with non-producers allowed the non-producers to share the benefits of DNA uptake.

Of course, questions also remain. Could GTAs also be beneficial to cells in other ways, or is DNA repair the single magic answer? The GTAs in this system were only produced after loss of function of a regulatory gene—how does this relate to the natural world where these bacteria exist? Do these laboratory experiments reflect the reality of the natural world? Is this a primary function and benefit of other GTAs in other organisms? The *C*. *crescentus* GTAs do not appear to have tails and are missing key tail protein genes—is the absence of observed tails a methodological artefact or can they really function for DNA transfer without a tail? This last point has implications beyond GTA research and could be important in the context of phage evolution.

Many other aspects about GTAs also warrant further investigation. Can GTAs be manipulated to be used as more broadly applicable tools? They are already used in lab settings to genetically manipulate the producing organism (e.g., as in [2]) and have properties that could make them useful beyond this. A high-resolution structure of RcGTA is now available [10] that includes important details about proteins involved in interactions with recipient cells and perhaps this information can be used to manipulate the particles’ “host range” such that they will transfer DNA to different species. Also, as mentioned above, RcGTA-transferred DNA enters the cell’s cytoplasm as ssDNA and therefore bypasses restriction enzyme defenses, which could allow DNA transfer to some otherwise recalcitrant species. GTAs will package any DNA so the development of an *in vitro* assembly system could allow the generation of GTAs containing targeted DNA regions for subsequent transfer. Additionally, is it possible to determine the abundance of GTA particles within natural environments and what we currently think of as “viral” communities? Analyses of genome sequences have revealed the high prevalence of prophages and viral-related genes in bacteria and archaea. It seems possible that some of these could be GTAs and experimental work by microbiologists with their favorite organisms could be done to look for evidence of GTA activity. This would broaden our understanding of the scope of GTA relevance in microbiology and evolution.
